# In-plane coherent control of plasmon resonances for plasmonic switching and encoding

**DOI:** 10.1038/s41377-019-0134-1

**Published:** 2019-02-06

**Authors:** Liyong Jiang, Tingting Yin, Alexander M. Dubrovkin, Zhaogang Dong, Yuntian Chen, Weijin Chen, Joel K. W. Yang, Zexiang Shen

**Affiliations:** 1 Department of Physics, School of Science, Nanjing University of Science and Technology, Nanjing, 210094 China; 2 Centre for Disruptive Photonic Technologies, The Photonics Institute, School of Physical and Mathematical Sciences, Nanyang Technological University, 21 Nanyang Link, Singapore, 637371 Singapore; 3 Institute of Materials Research and Engineering, A*STAR (Agency for Science, Technology and Research), #08-03 Innovis, Singapore, 138634 Singapore; 4 School of Optical and Electronic Information, Huazhong University of Science and Technology, Wuhan, 430074 China; 5 Singapore University of Technology and Design, 8 Somapah Road, Singapore, 487372 Singapore

**Keywords:** Nanophotonics and plasmonics, Nanoparticles

## Abstract

Considerable attention has been paid recently to coherent control of plasmon resonances in metadevices for potential applications in all-optical light-with-light signal modulation and image processing. Previous reports based on out-of-plane coherent control of plasmon resonances were established by modulating the position of a metadevice in standing waves. Here we show that destructive and constructive absorption can be realized in metallic nano-antennas through in-plane coherent control of plasmon resonances, which is determined by the distribution rule of electrical-field components of nano-antennas. We provide proof-of-principle demonstrations of plasmonic switching effects in a gold nanodisk monomer and dimer, and propose a plasmonic encoding strategy in a gold nanodisk chain. In-plane coherent control of plasmon resonances may open a new avenue toward promising applications in optical spectral enhancement, imaging, nanolasing, and optical communication in nanocircuits.

## Plasmonic devices: Better control with in-plane light

Shining light along metal surfaces could provide unprecedented control of plasmons for optical devices, say researchers in China and Singapore. Localized surface plasmon resonances (LSPRs) are oscillations in conduction electrons coupled with light photons, which could act as information carriers for nano-sized optical sensors or even computers. Most attempts to control LSPRs use “out-of-plane” light incident at right angles to surfaces; however “in-plane” illumination—shining light at low angles across surfaces—also holds promise. Liyong Jiang and co-workers at Nanjing University of Science and Technology have demonstrated two new methods for in-plane illumination of LSPRs on gold nanodisks. They showed that they could not only switch the LSPRs into different states by adjusting the incident light, but also encode logical data onto chains of nanodisks, in ways that were not possible with out-of-plane illumination.

## Introduction

Over the past few years, significant efforts have been devoted to studying the strong light–matter interactions in plasmonic systems at nanoscale^1^. Based on the control of localized surface plasmon resonance (LSPR), many practical applications have been reported, including surface-enhanced Raman scattering^2,3^, plasmon waveguides^4^, molecular rulers^5^, biosensing and bioimaging^6,7^, surface-enhanced fluorescence^8,9^, nanolasers^10–12^, plasmonic color printers^13,14^, plasmonic tunnel junctions^15^, and plasmonic holography and metalens^16–18^. In these pioneering works, the control of plasmon resonances focused on designing the configurations of plasmonic nanostructures. People already understood the size- and shape-dependent^19,20^ LSPR of single plasmonic nanoparticles and coupled plasmonic systems^21,22^ based on the classical Mie theory^23^ and well-established plasmonic hybridization models^24–26^. The resonant wavelength of the fundamental LSPR is proportional to the single nanoparticle’s size, which is below the quasi-static approximation limitation. The bright and dark plasmon resonances, as well as the Fano-like^27^ and electromagnetically induced transparency^28^ phenomena in complex plasmonic systems, are determined by the LSPR coupling and energy transfer.

Moreover, in conventional optical studies of single and coupled nano-antennas, the light beam usually illuminates normally to the sample surface from one direction. As a result, the control of plasmon resonances can also be realized under asymmetric out-of-plane illumination conditions by changing incident light parameters, including polarization, amplitude, and phase, or by using a pulsed-beam and single-photon source for ultrafast^29^, nonlinear^30^, and quantum optical study^31^. Recently, coherent control of plasmon resonances under symmetrical out-of-plane illumination has opened a new way of signal modulation. By changing the position of a metadevice in standing waves, interaction between light and the metadevice can reach the maximum and minimum at the antinode and node, respectively. Based on an out-of-plane interferometric setup, coherent perfect absorption^32,33^ and transparency in plasmonic metasurfaces have been demonstrated to show novel applications in optical communication, such as all-optical light-with-light coherent modulators^34^, optical amplifiers^35^, and arithmetic units^36^. However, the out-of-plane coherent control of plasmon resonances has shown obvious limitations in mode and spatial selection. For example, optical response is generally uniform for nano-antennas on the metadevice due to isotropy-coherent absorption in the sample plane normal to the incidence.

Here we first report a study on in-plane coherent control of plasmon resonances in typical metallic nano-antennas. It has been widely reported that for nano-antennas, the plasmon resonances at oblique incidence^37–40^ are quite different from those under normal incidence due to the retardation effect^41^, which indicates anisotropy optical response in the sample plane under a symmetric in-plane illumination condition. We show selectively multimode coherent absorption in a gold nanodisk monomer and dimer, as well as spatial selection of coherent absorption in a gold nanodisk chain. We provide proof-of-principle demonstrations of plasmonic switching and encoding applications based on the mode and spatial selection of coherent absorption in single and coupled gold nanodisks.

## Results

### Setups for in-plane coherent control of plasmon resonances

**Fig. 1:**
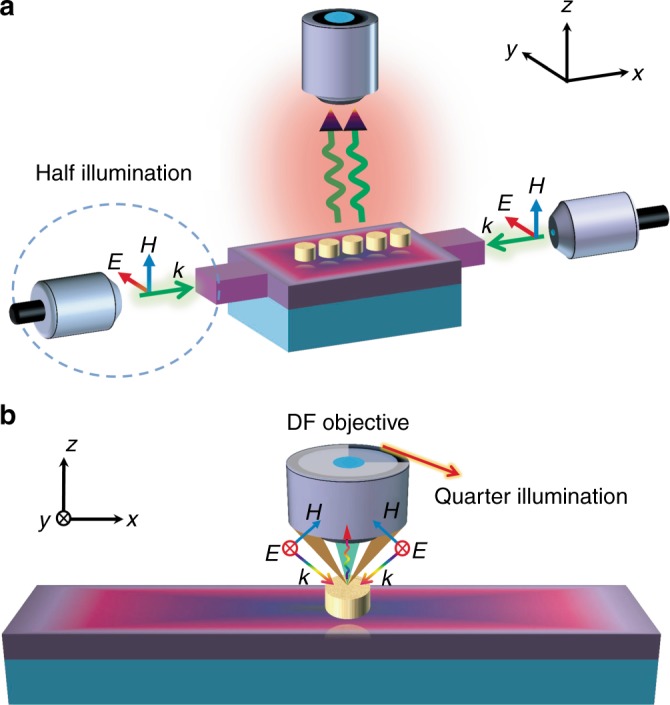
Schematic diagrams of two setups for in-plane coherent control of plasmon resonances. **a** Fiber-waveguide interferometer. **b** Dark-field (DF) confocal microscope, where quarter illumination can be satisfied by blocking 3/4 area of the annular aperture

Two possible setups are proposed here for in-plane coherent control of plasmon resonances through changing the symmetry of in-plane illumination. One is based on a fiber-waveguide interferometer (Fig. 1a), where completely symmetrical in-plane illumination is constructed by coupling two polarized coherent beams from the single-mode fiber-connected objective lens into the input waveguides without phase delay, whereas asymmetrical in-plane illumination can be realized by blocking one input beam (half-illumination) or introducing a phase delay between two beams. This setup can be widely used to study the in-plane coherent control of plasmon resonances in all kinds of plasmonic nanostructures with axial symmetry. The out-of-plane far-field signal carrying the LSPR information will be collected by an objective lens. However, this kind of setup still faces challenges in experiments, including accurate phase control required between two input fibers and high coupling efficiency required between fibers and waveguides. As a comparison, the widely used dark-field (DF) confocal microscopy is more convenient (Fig. 1b), where the polarized light is focused onto the sample through a condenser lens with an annular aperture and the backward scattering light is collected by an objective lens in a confocal setup. In this setup, completely symmetrical in-plane illumination can be easily satisfied once the input light is focused onto the center of the sample, whereas asymmetrical in-plane illumination (e.g., quarter illumination) can be constructed by blocking three-fourth of the area of the annular aperture. It is clear that such a DF illumination setup still contains out-of-plane wave vector components; thus, a relatively large numerical aperture (NA) of the condenser lens is required to reach the grazing incident condition. This setup is suitable for studying plasmonic nanostructures with sizes comparable to the focused spot size of the incident light beam.

### In-plane coherent control of plasmon resonances with destructive and constructive interference

**Fig. 2:**
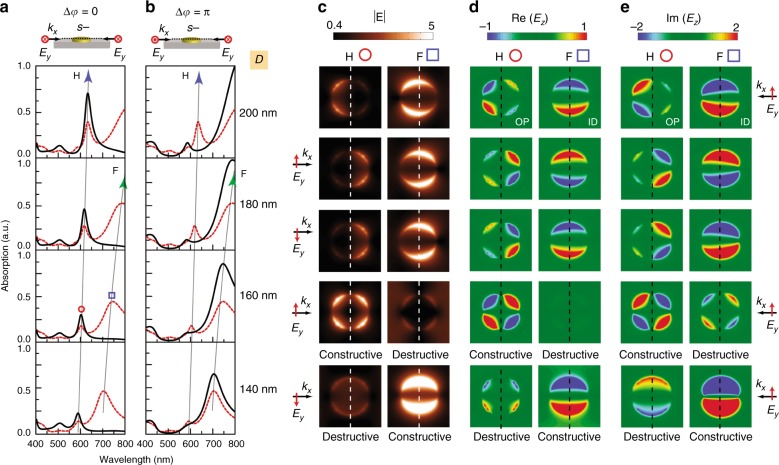
In-plane coherent control of plasmon resonances in gold nanodisk monomers. **a**, **b** Calculated normalized absorption spectra of gold nanodisk monomers with a diameter ranging from 140 to 200 nm for *s*-polarized in-plane plan wave coming from the right side (dashed line) or both sides (solid line) without phase delay, or with a phase delay of *π*. “F” and “H” represent fundamental and high-order plasmon resonances. **c**–**e** The corresponding spatial distributions of electric-field amplitude |*E*|, real part Re(*E*_z_), and imaginary part Im(*E*_z_) for the “F” and “H” modes (square and circle signs) of the representative gold nanodisk monomer (*D* = 160 nm) under asymmetrical and symmetrical in-plane illumination. Under symmetrical in-plane illumination, we can observe phase delay-dependent destructive/constructive interference for the “F” and “H” modes

Based on the fiber-waveguide interferometer, we theoretically studied the in-plane coherent control of plasmon resonances in gold nanodisks. Figure 2a, b shows the calculated absorption spectra of gold nanodisk monomers on SiO_2_/Si substrate with diameter ranging from 140 to 200 nm. For each gold nanodisk, the *s*-polarized plan wave was introduced from the right side (dashed line), generating one fundamental LSPR (“F”) peak and one retardation-induced high-order plasmon resonance (“H”) peak, both of which continuously red-shift with increasing diameters of gold nanodisks^41^. When symmetrical in-plane illumination (solid line) was applied without phase delay, the “F” mode was weakened and the “H” mode was apparently enhanced^42^. As a comparison, when a phase delay of *π* was applied in the symmetrical in-plane illumination, an opposite weakened/enhanced interference was observed for the two modes.

The weakened and enhanced phenomena are caused by the destructive and constructive plasmon resonances, which can be clearly demonstrated by the spatial distributions of electric-field amplitude for the gold nanodisk with a diameter of 160 nm (Fig. 2c and Supplementary Movie 1). The destructive and constructive interference can be further explained by the distributions of real and imaginary parts of electric-field component *E*_z_ for the “F” and “H” modes (Fig. 2d, e). More specifically, when *s*-polarized light comes only from the right side, the corresponding Re(*E*_z_) and Im(*E*_z_) distributions are identical (“ID”) and opposite (“OP”) for the “F” and “H” modes, respectively. Considering that the nanodisk is axisymmetric, when *s*-polarized light comes from the left side without phase delay, the corresponding *E*_z_ distribution will be mirrored with a negative sign along the central axis of the nanodisk (*x* = 0), i.e., *E*_z_(− *x*) = − *E*_z_(*x*). As a result, the “F” mode with “ID” Re(*E*_z_) and Im(*E*_z_) distribution caused by right-side illumination will cancel with its negative mirror caused by left-side illumination and result in destructive interference, whereas the “H” mode with “OP” Re(*E*_z_) and Im(*E*_z_) distribution will superpose with its negative mirror and lead to constructive interference. As a comparison, when *s*-polarized light comes from the left side with a phase delay of *π*, the result of using a mirror operator is *E*_z_(− *x*) = *E*_z_(*x*). As a result, under symmetrical illumination with a phase delay of *π*, the “F” and “H” modes will experience constructive and destructive interference, respectively. For the case of *p*-polarized light, the phase delay-dependent mirror operators are consistent with the *s*-polarized light under symmetrical illumination. However, when *p*-polarized light comes only from the right side, the corresponding Re(*E*_z_) and Im(*E*_z_) distributions are opposite (“OP”) and identical (“ID”) for the “F” and “H” modes, respectively. Therefore, the total phase delay-dependent destructive/constructive interference for the “F” and “H” modes are opposite to the case of *s*-polarization (Fig. S1 in Supplementary Information). The destructive/constructive interference of plasmon resonances was rechecked by the electromagnetic multipole theory, based on which the calculated absorption spectra of a 200nm gold nanodisk monomer under symmetrical illumination matched well with the simulation results (Fig. S2 and Supplementary Note 1 in Supplementary Information). We have also studied the in-plane coherent control of plasmon resonances in gold nanodisk dimers, which show opposite phase delay-dependent destructive/constructive interference for the “F” and “H” modes as compared with the monomers (Fig. S3 in Supplementary Information).

### Demonstration of electric-field distribution rule by s-SNOM

**Fig. 3:**
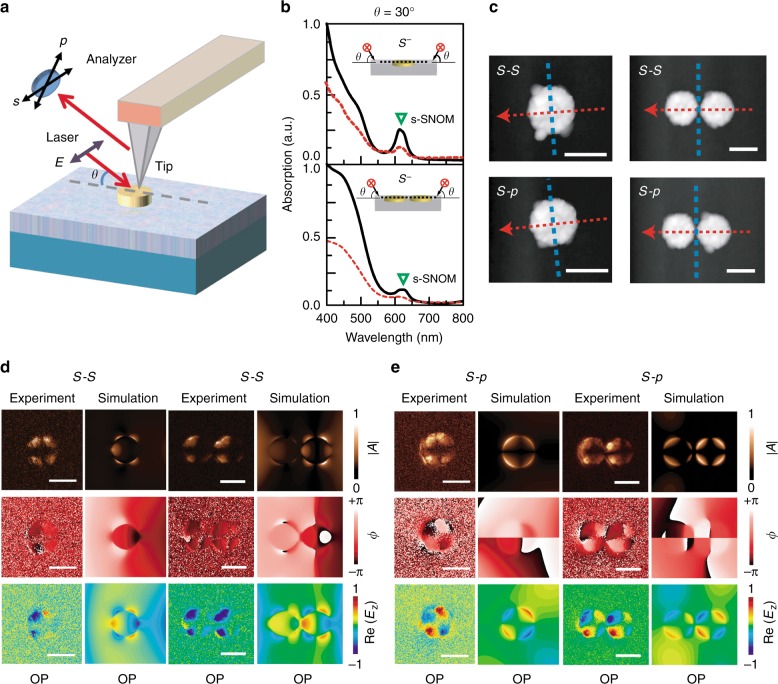
Demonstration of electrical-field distribution rule for the 200 nm gold nanodisk monomer and dimer by s-SNOM. **a** Schematic of the s-SNOM measurement for *s*–*s* and *s*–*p* excitation–collection configurations. The wavelength of the excitation laser is 633 nm and the incidence angle with respect to the plane of the substrate is 30°. **b** Calculated normalized absorption spectra of 200 nm gold nanodisk monomer and dimer at incidence angle 30° under asymmetrical (dashed line) or symmetrical (solid line) illumination without phase delay. The gap size in the dimer is 30 nm. **c** Atomic-force microscopic (AFM) images of gold nanodisk monomer and dimer for *s*–*s* and *s*–*p* measurements. The red arrow represents the incidence direction of the laser and the blue dashed line represents the central axis of the nanodisk. **d**, **e** Experimental and simulated spatial distributions of the amplitude |A|, phase ϕ, and real part of electric-field component *E*_y_ in *s*–*s* measurement and *E*_z_ in *s*–*p* measurement for 200 nm gold nanodisk monomer and dimer. The scale bar is 200 nm

Table 1 summarizes the distribution rule for all three electrical-field components under in-plane symmetric illumination, where the rule of destructive/constructive interference is always identical for *E*_y_ and *E*_z_, but opposite for *E*_x_. We used the polarization-sensitive scattering-type scanning near-field optical microscopy (s-SNOM) to verify the electric-field distribution rule. The s-SNOM measurement was conducted by a focused laser (*λ* = 633 nm) coming from one side with an incidence angle of 30° with respect to the plane of the substrate, corresponding to an *s*–*s*/*s*–*p* geometry scheme (Fig. 3a). For *s*–*s* measurement, we recorded *E*_*s–s*_ which is equal to *E*_y_. In *s*–*p* measurement, we detected the total electric field *E*_*s–p*_, written by (*E*_x_^2^ + *E*_z_^2^)^1/2^, where the calculated amplitude of *E*_z_ is much larger than *E*_x_ and thus the total electric field *E*_*s–p*_ is roughly equal to *E*_z_. As an example, *s*–*s* and *s*–*p* excitation–collection measurements were carried out on a 200 nm gold nanodisk monomer and dimer with a gap size of 30 nm. From Fig. 3b, we can find that the “H” mode at incidence angle 30° is located near the excitation wavelength, and both the monomer and dimer show constructive interference of “H” mode under symmetrical illumination without phase delay, which implies that both Re(*E*_y_) and Re(*E*_z_) should satisfy an “OP” distribution, according to Table 1. Figure 3d shows the measured and simulated amplitude, phase, and real part of *E*_y_ for the *s*–*s* excitation–collection measurement, whereas the corresponding results of the *E*_z_ component for *s*–*p* measurement are shown in Fig. 3e. Both measured and simulated results give strong evidence to demonstrate the “OP” distribution for both Re(*E*_y_) and Re(*E*_z_), where single and coupled quadrupole-like plasmon resonance can be observed for the monomer and dimer, respectively.

**Table 1:** Electric-field distribution rule for destructive and constructive plasmon resonances under symmetrical in-plane illumination

Phase delay	*s*-/*p*-polarization	Re/Im(*E*_x_)	Re/Im(*E*_y_)	Re/Im(*E*_z_)
0	Destructive	OP	ID	ID
0	Constructive	ID	OP	OP
*π*	Destructive	ID	OP	OP
*π*	Constructive	OP	ID	ID

### Plasmonic switching in gold nanodisk monomer and dimer

**Fig. 4:**
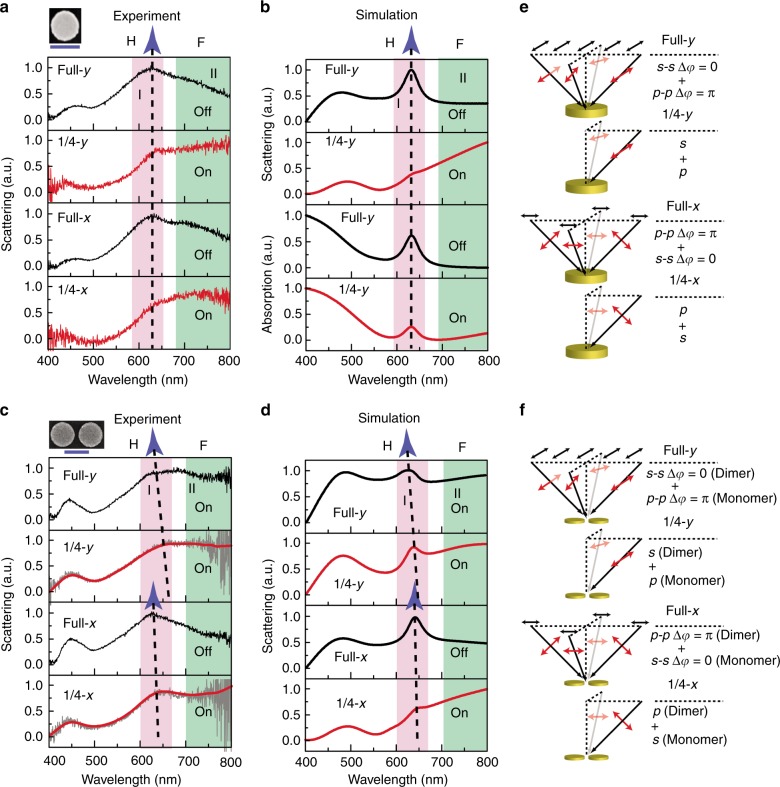
Demonstration of plasmonic switching by DF scattering measurement of gold nanodisk monomer and dimer. **a** Normalized DF scattering spectra of gold nanodisk monomer with a diameter of 200 nm (SEM image) under full and quarter illumination. **b** The corresponding normalized simulated scattering and absorption spectra. **c**, **d** Normalized measured and simulated DF scattering spectra of gold nanodisk dimer with a diameter of 200 nm and a gap size of 30 nm (SEM image) under full and quarter illumination. The red solid curves in Fig. 4c are the smoothing results. The scale bar in SEM images is 200 nm. **e**, **f** Polarization diagrams of full and quarter illumination in the DF scattering measurement and simulation for gold nanodisk monomer and dimer. In both experiment and simulation, the excitation is *s*- or *p*-polarized and the collection is unpolarized. The black and red double-headed arrows represent the initial polarization and the polarization after focusing, respectively

One promising application based on the in-plane coherent control of plasmon resonances is plasmonic switching. Here we employed the DF confocal microscopy (Fig. 1b) to experimentally demonstrate the plasmonic switching effect in a gold nanodisk monomer and dimer. During the measurement, a condenser lens with a high NA (0.9) was used to provide sufficient in-plane illumination and an opaque tape was used to construct the quarter illumination. Both *s*- and *p*-polarization components of excitation were considered, and the collection was unpolarized. We experimentally observed the plasmonic switching effect in a 200 nm gold nanodisk monomer (Fig. 4a), i.e., constructive interference (area I) for the “H” plasmon resonance and destructive interference (area II) for the “F” plasmon resonance. Such a plasmonic switching phenomenon is clear and consistent for both *y*- and *x*-polarization under full and quarter illumination, and is in good agreement with the simulated scattering and absorption spectra (Fig. 4b). The polarization diagrams for full and quarter illumination (Fig. 4e) indicate that the *y*- and *x*-polarization of excitation are identical for the gold nanodisk monomer, resulting in an isotropic plasmonic switching effect. As a comparison, the plasmonic switching effect only occurs for the *x*-polarization of excitation in the gold nanodisk dimer, as shown in Fig. 4c, d. The anisotropic plasmonic switching effect in the gold nanodisk dimer is due to the superposition of anisotropic destructive and constructive plasmon resonances contributed by the in-plane wave vectors along the long and short axis, as shown in Fig. 4f.

### Plasmonic encoding in gold nanodisk chains

**Fig. 5:**
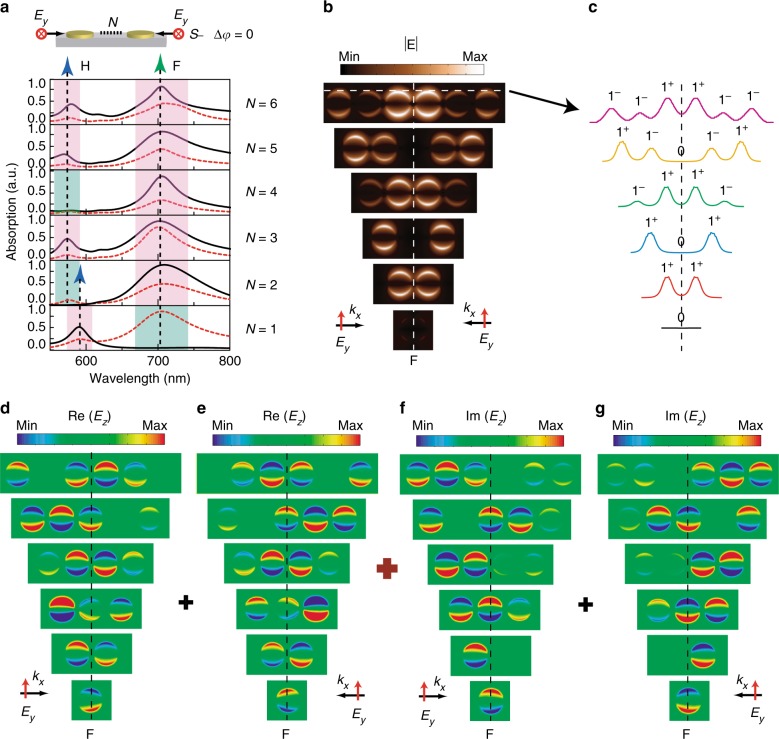
Plasmonic encoding in gold nanodisk chains. **a** Calculated absorption spectra of gold nanodisk chains consisting of different numbers of nanodisks illuminated by the *s*-polarized in-plane plan wave coming from right side (dashed line) or both sides (solid line). The diameter of the nanodisk is 140 nm and the separation distance is 30 nm. The destructive and constructive plasmon resonances are represented by green and red colors, respectively. **b** Spatial distributions of electric-field amplitude |E| for the “F” plasmon resonances (peak position) under symmetrical illumination. **c** Sliced electric-field amplitude distributions along the chain’s edge (the white dashed line in Fig. 5b). **d**–**g** Corresponding spatial distributions of real and imaginary part of *E*_z_ when the *s*-polarized in-plane plan wave comes from the left side (**d**, **f**) and right side (**e**, **g**) respectively

Another possible application based on the in-plane coherent control of plasmon resonances is plasmonic encoding. Here we propose a plasmonic encoding strategy in a series of gold nanodisk chains consisting of different numbers of 140 nm nanodisks with a separation distance of 30 nm. Figure 5a shows the absorption spectra of gold nanodisk chains under in-plane asymmetric (dashed line) or symmetrical (solid line) *s*-polarized illumination without phase delay. The “F” mode for gold nanodisk chains with number *N* from 2 to 6 always shows constructive interference under the symmetrical illumination, whereas the “H” mode shows alternate constructive and destructive interference from nanodisk monomer to dimer, trimer, tetramer, and pentamer. Figure 5b shows the spatial distributions of electric-field amplitude for the “F” mode under symmetrical illumination, where the gold nanodisk chains show quite different propagating coupling behavior compared with that under asymmetric illumination. More specifically, under asymmetric illumination, the plasmonic coupling and propagation are along the long axis of the gold nanodisk chain and decrease in intensity (Fig. S4 and Supplementary Note 2 in Supplementary Information)^42^. Under symmetrical illumination, the plasmonic coupling and propagation are no longer continuous. The nanodisk in the center of chains with odd numbers of *N* always shows completely destructive interference, whereas the two nanodisks in the center of chains with even numbers of *N* always show constructive interference (see Supplementary Movie 2). The spatially related destructive and constructive interference inside the gold nanodisk chains can be used for plasmonic encoding, as shown in the sliced electric-field amplitude distributions in Fig. 5c, where signals 0, 1^−^, and 1^+^ can be generated based on the relative intensity along the nanodisk chain’s edge. The plasmonic encoding can be well explained by the superposition of Re(*E*_z_) and Im(*E*_z_) distributions under left- and right-side illumination, as shown in Fig. 5d-g, where the destructive and constructive interference always occurs when the nanodisk satisfies “OP” and “ID” distributions, respectively. Besides the number of gold nanodisk chains, the input phase delay can also affect the plasmonic encoding. The signals 0 and 1^+^ inside the gold nanodisk pentamer can be step-by-step shifted to the adjacent nanodisks by introducing an increasing phase delay from 0 to 3*π*/2, with an interval of *π*/2 (Fig. S5 and Supplementary Movie 3).

## Discussion

In summary, we have demonstrated the idea of coherent control of plasmon resonances under symmetrical in-plane illumination. The coherent absorption for a single gold nanodisk shows a distinct mode-selection character due to destructive/constructive fundamental and higher-order plasmon resonances. We established and experimentally verified the distribution rule of electrical-field components for realizing destructive and constructive plasmon resonances in an axisymmetric plasmonic nanostructure. The constructive fundamental plasmon resonance for a gold nanodisk chain shows a distinct spatial-selection character due to phase-dependent coherent absorption along the chain. Compared with out-of-plane coherent control, in-plane coherent control of plasmon resonances strongly relies on the configuration and symmetry of plasmonic nanostructures rather than the spatial position of metadevices placed in standing waves. This allows us more freedom in tailoring and engineering the multiple plasmon resonances in other axisymmetric plasmonic nanostructures, including nanosphere, nanorod, nano bowtie, and nanostructure polymer.

Besides the proof-of-principle demonstrations of plasmonic switching and encoding, more potential applications based on in-plane coherent control of plasmon resonances can be expected. Specifically, the plasmonic switching effect is ready to be extended to the study of selective surface-enhanced spectra, such as surface-enhanced fluorescence^43^ and surface-enhanced Raman scattering^44^, during which the photoluminescence or Raman signal of multiple molecules can be selectively enhanced by controlling the on/off state of multiple plasmon resonances in one common nano-antenna^45^. A plasmonic encoding scheme can be extended to plasmonic imaging, nanolasing, and optical communication in nanocircuits. For instance, plasmonic nanostructure arrays doped with different fluorophore or gain materials can be used to realize selective plasmonic imaging^46^ or nanolasers^12^ through spatially selective coherent absorption in the array. By using combined plasmonic nanostructure chains with different encoding characteristics, logic units (such as XOR, NOT, and AND) and multichannel waveguides can be designed for all-optical information storage and processes^35,36^.

## Materials and methods

### Fabrication of gold nanodisks

We employed the electron-beam lithography (EBL) and a lift-off process to fabricate the gold nanodisk samples on SiO_2_/Si substrates^47^, where a 100 nm-thick SiO_2_ layer was used for reducing plasmon damping. The gold film (30 nm thick) and the underlying Cr adhesion layer (1 nm thick) were deposited on the SiO_2_/Si substrate through the electron-beam evaporation. During the EBL process (Elionix ELS-7000), the accelerating voltage and beam current were set to 100 keV and 100 pA, respectively. *N*-methylpyrrolidone solvent was used in the lift-off process and the solvent was heated up to 65 °C .

### s-SNOM imaging

A polarization-sensitive s-SNOM was used to image the plasmon resonance modes in gold nanodisks based on a Neaspec commercial instrument, where a balanced technique was implemented for the optical-signal detection^48^. We used an *s*–*s*/*s*–*p* geometry scheme and engaged a dielectric (Si) tip for measurements, during which *s*-polarized CW laser radiation (*λ* = 633 nm) was used to illuminate the sample with an incidence angle of 30° with respect to the plane of the substrate. The plasmonic mode images were recorded simultaneously with the disk’s topography upon raster scanning of the sample. Here, the tip-scattered optical signal and tip-height position were recorded at each point of the scan. The amplitude and phase of the scattered signal were measured based on the pseudoheterodyne interferometric detection at the fourth harmonic of the tip-tapping frequency. An analyzer placed in front of the detector was used to select the *p*- or *s*-polarized component of the scattered light.

### DF scattering measurement

A confocal Raman microscopy system (WITec CRM200) was used to measure the DF scattering spectra of gold nanodisk samples. A high-power halogen lamp (Philips, 100 W) was used as the light source. The scattering light was collected by a × 100 DF objective lens (Zeiss Epiplan, NA = 0.9) and the final spectra was generated by a TE-cooled charge-coupled device (Andor DV 401-BV-351) with a 150 line/mm grating in front of it. In the quarter-illumination measurement, the input of a condensed DF lens was blocked in three-fourth area by an opaque tape. In all measurements of scattering spectra, the integration time was 20 s.

### Numerical simulations

We employed a commercial software package (Lumerical FDTD Solutions) to simulate the electrical-field distributions, absorption, and scattering spectra of the gold nanodisks. For *x*, *y*, and *z* directions, the boundary conditions were set to perfectly matched layer and the finest mesh size in the structure was set to 1 nm. In Figs. 2 and 5, the simulation was done with a pure SiO_2_/Si substrate, whereas the simulation also contained the Cr adhesion in Figs. 3 and 4. The complex electromagnetic parameters were from Johnson and Christy for Au and Cr, and from Palik for SiO_2_ and Si^49^.

## Supplementary information

Supplementary Information

Supplementary Movie 1

Supplementary Movie 2

Supplementary Movie 3
