# Supplementary material for: In-plane coherent control of plasmon resonances for plasmonic switching and encoding
Source: Light Sci Appl. 2019 Feb 6;8:21. doi: 10.1038/s41377-019-0134-1 (PMC6363765; doi:10.1038/s41377-019-0134-1)
Supplement: Supplementary file 1 — Supplementary Information [file 41377_2019_134_MOESM1_ESM.docx]

Supplementary Information for

In-plane coherent control of plasmon resonances for plasmonic switching and encoding

Liyong Jiang^1,2,*^, Tingting Yin^2^, Alexander M. Dubrovkin^2^, Zhaogang Dong^3^, Yuntian Chen^5^, Weijin Chen^5^, Joel K. W. Yang^3,4,*^, and Zexiang Shen^2,*^

*^1^Department of Physics, School of Science, Nanjing University of Science and Technology, Nanjing 210094, China.*

*^2^Centre for Disruptive Photonic Technologies, The Photonics Institute, School of Physical and Mathematical Sciences, Nanyang Technological University, 21 Nanyang Link, Singapore 637371.*

*^3^Institute of Materials Research and Engineering, A*STAR (Agency for Science, Technology and Research), #08-03 Innovis, Singapore 138634.*

*^4^Singapore University of Technology and Design, 8 Somapah Road, Singapore 487372.*

*^5^School of Optical and Electronic Information, Huazhong University of Science and Technology, Wuhan 430074, China.*

**Corresponding author:* [*jly@njust.edu.cn*](mailto:jly@njust.edu.cn)*;* [*joel_yang@sutd.edu.sg*](mailto:joel_yang@sutd.edu.sg)*;* [*zexiang@ntu.edu.sg*](mailto:zexiang@ntu.edu.sg)


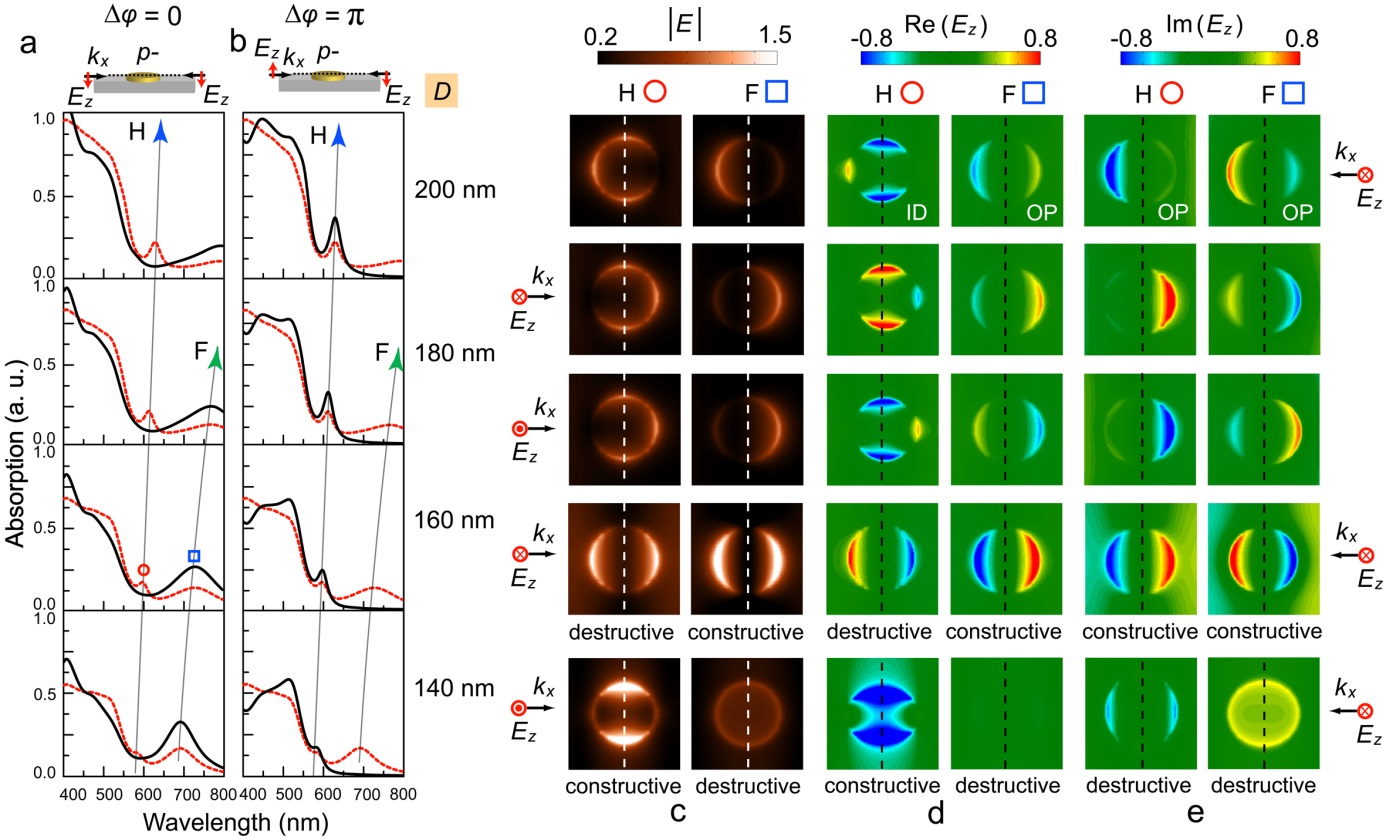


**Fig. S1** **In-plane coherent control of** **plasmon resonances in gold nanodisk** **monomers (*p*-polarization).** **a,b** Calculated normalized absorption spectra of gold nanodisk monomers with a diameter ranging from 140-200 nm for the *p*-polarized in-plane plan wave coming from the right side (dashed line) or both sides (solid line) without phase delay or a phase delay of π. ‘F’ and ‘H’ represent the fundamental and high-order plasmon resonances. **c**-**e** The corresponding spatial distributions of electric field amplitude |*E*|, real part Re(*E_z_*), and imaginary part Im(*E_z_*) for the ‘F’ and ‘H’ modes (square and circle signs) of the representative gold nanodisk monomer (*D*=160 nm) under asymmetrical and symmetrical in-plan illumination.


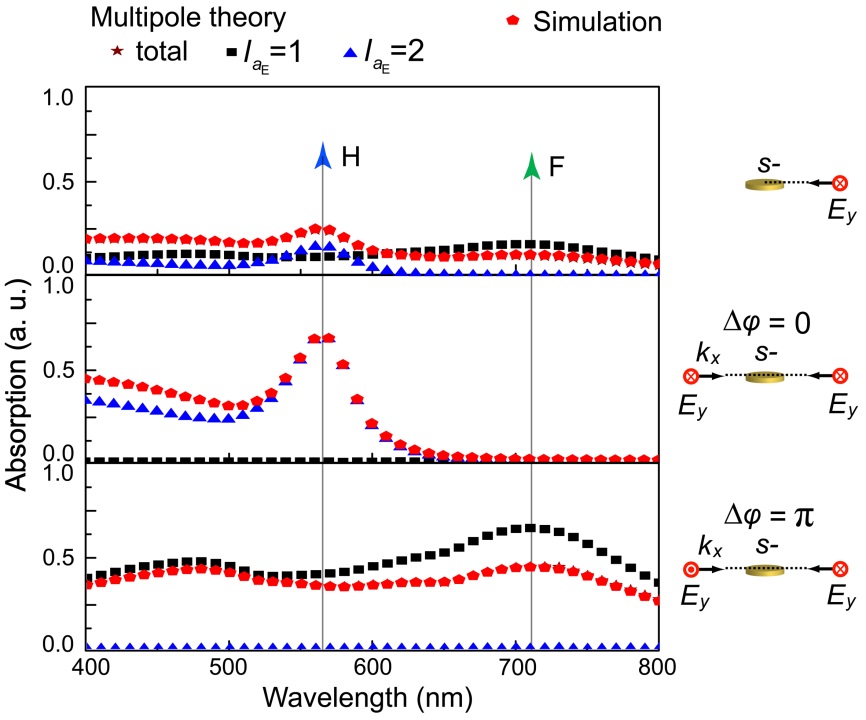


**Fig. S2** **Verifying** **phase delay dependent destructive/constructive interference** **of plasmon resonances based on the electromagnetic multipole theory.** Calculated normalized absorption spectra of a pure 200-nm gold nanodisk monomer when the asymmetrical/symmetrical *s*-polarized plan wave comes from the right side or both sides without phase delay or a phase delay of π. More details about the electromagnetic multipole theory are available in Supplementary Note 1.


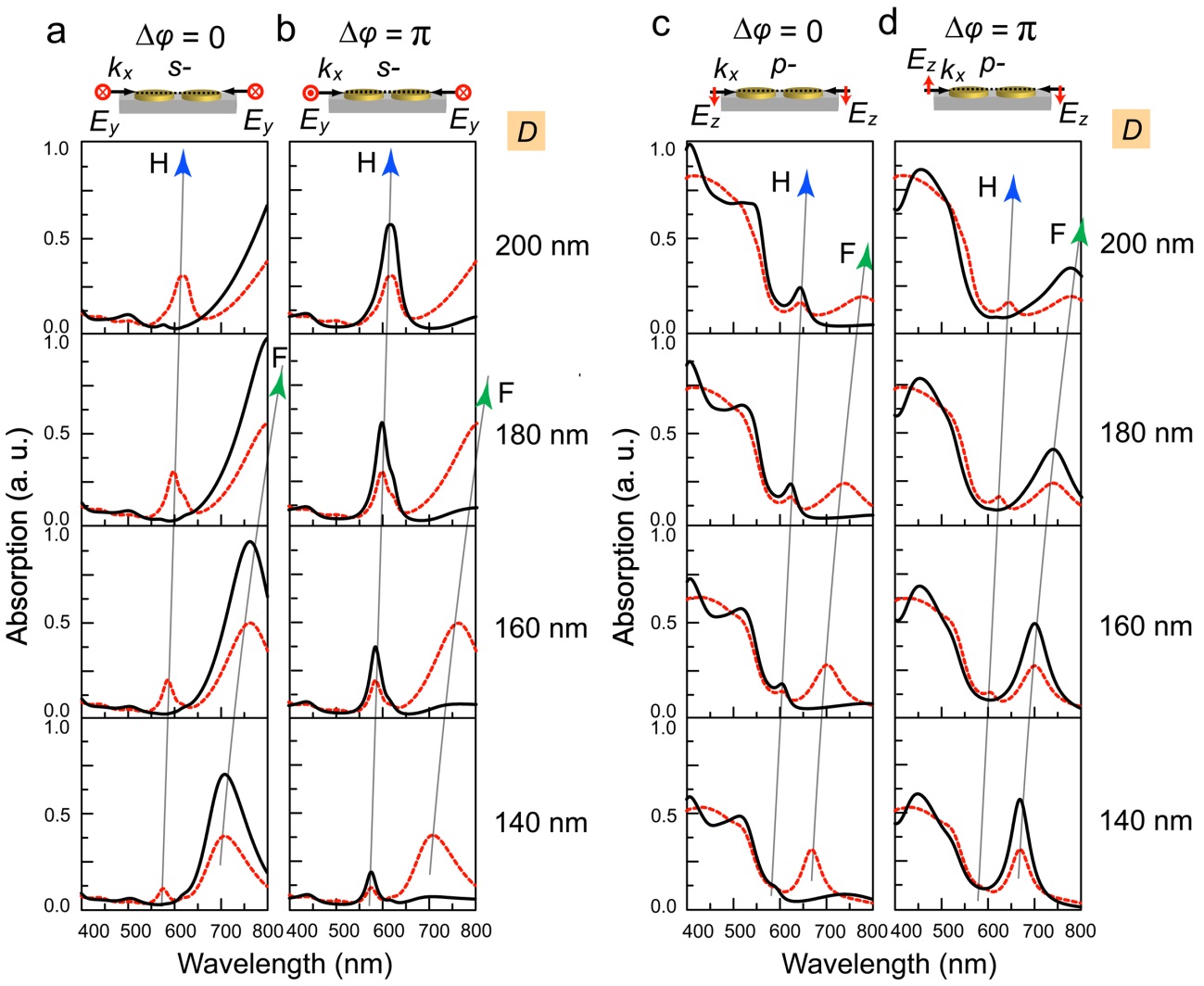


**Fig. S3** **In-plane coherent control of plasmon resonances in gold nanodisk dimers.** Calculated normalized absorption spectra of gold nanodisk dimers with a diameter ranging from 140-200 nm and a separation distance of 30 nm when the *s*- (**a,b)** or *p*-polarized (**c,d**) in-plane plan wave comes from the right side (dashed line) or both sides (solid line) without phase delay or a phase delay of π.

**
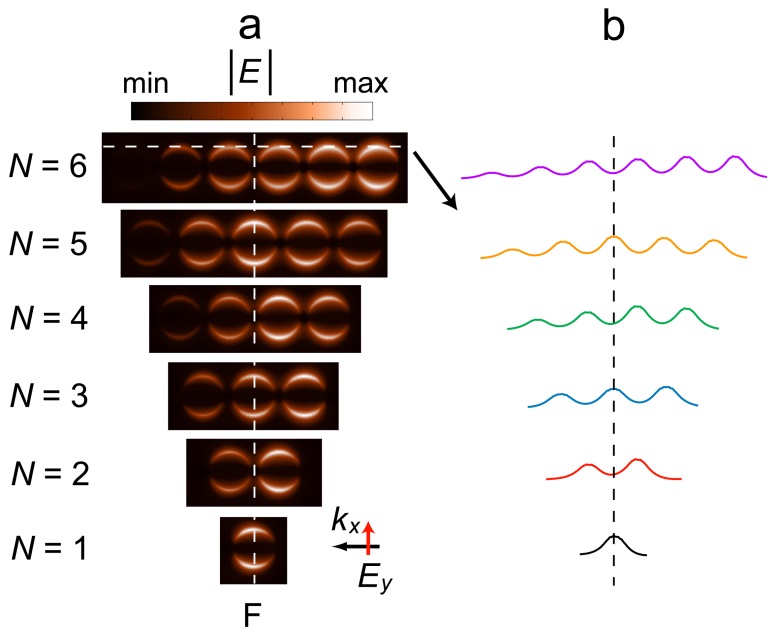
**

**Fig. S4** **Plasmonic coupling in gold nanodisk chains under asymmetrical illumination.** Spatial distributions of electric field amplitude |*E*| for the fundamental plasmon resonances in different gold nanodisk chains when the *s*-polarized in-plane plan wave comes from the right side.

**
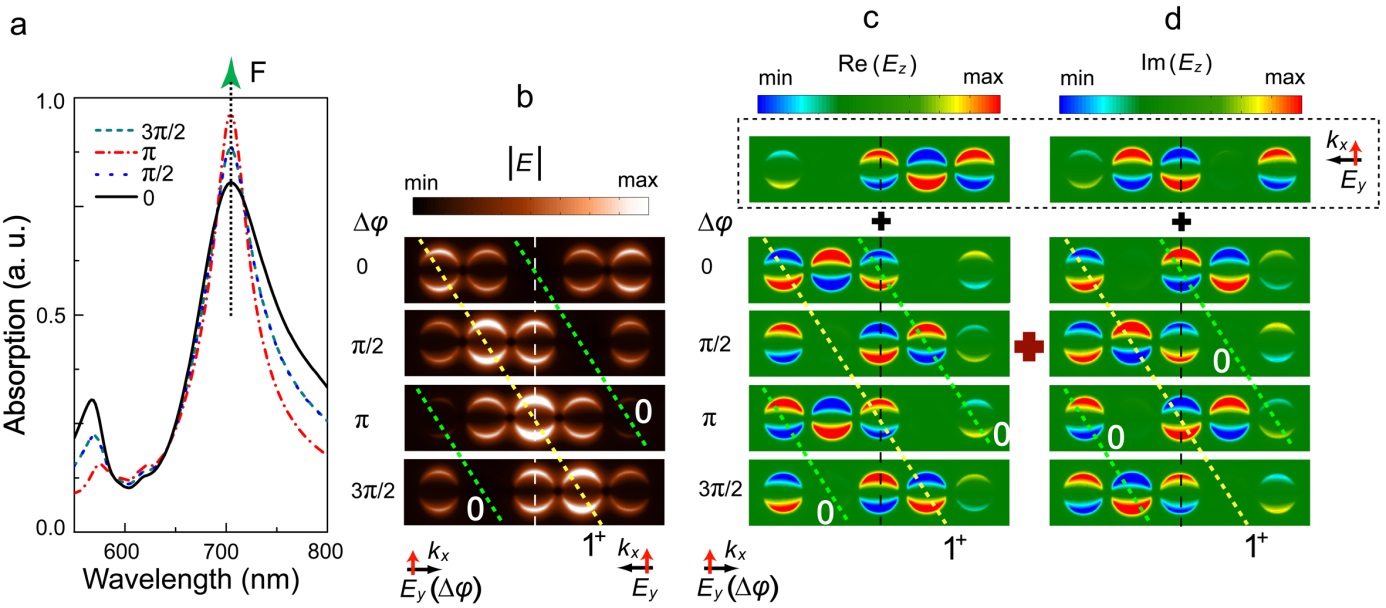
**

**Fig. S5 Phase-delay dependent plasmonic encoding in a gold nanodisk chain. a** Calculated normalized absorption spectra of the gold nanodisk pentamer when the *s*-polarized plan wave comes from both sides with different phase delays. **b** Spatial distributions of electric field amplitude |*E*| for the ‘F’ plasmon resonance in Fig. S5**a** under symmetrical illumination. Signals 0 and 1^+^ can be step-by-step shifted to the adjacent nanodisks when the phase delay is changing from 0 to 3π/2 with an interval of π/2. **c,d** The corresponding spatial distributions of Re(*E_z_*) and Im(*E_z_*) when the *s*-polarized plan wave comes from the right side (top panel) or left side (bottom panel) with different phase delays. The yellow and green dashed lines represent signals 0 and 1^+^, respectively. The phase-delay dependent plasmonic encoding in a gold nanodisk chain can be well explained based on the electric field distribution rule in Table 1.

**Supplementary Note 1 | Electromagnetic multipole theory under symmetrical illumination condition**

According to the electromagnetic multipole theory^1^, for a nanodisk, the extinction cross section for the *s*-polarized illumination can be written as:

$C_{\text{ext},s}=-\frac{\pi}{k^{2}}\sum_{l=1}^{\infty} \sum_{m=-1,+1} (2l+1)\mathrm{Im}\left[a_{\text{E}}(l,m)+ma_{\text{M}}(l,m)\right]$ (1)

$C_{\text{ext},p}=-\frac{\pi}{k^{2}}\sum_{l=1}^{\infty} \sum_{m=-1,+1} (2l+1)\mathrm{Re}\left[ma_{\text{E}}(l,m)+a_{\text{M}}(l,m)\right]$ (2)

where *k* is the wave number, *l* is the order of pole, and *m* is the angular quantum number. $a_{\text{E}}$ and $a_{\text{M}}$ are the electrical and magnetic multipole coefficients.

Under the symmetrical illumination condition, we derived the extinction cross section as:

(3)

(4)

Here is the mirror operator along the central axis of a nanodisk (*x*=0), i.e., $a_{\text{E}}(l,m)^{*}_{\mp x}=a_{\text{E}}(l,m)_{\pm x}$.

Figure S2 shows an example for a pure 200-nm gold nanodisk monomer under asymmetrical and symmetrical *s*-polarized illumination. The theoretical results based on equations (1) and (3) match well with the simulated results.

**Supplementary Note 2 | Light propagation in a metallic nanoparticle chain under asymmetric illumination**

Previous works^2-4^ have well demonstrated that under asymmetric illumination light can propagate in a metallic nanoparticle chain with close interparticle spacing through strong near-field LSPR interactions and thus can break the diffraction limitation. The propagation dispersion and propagation loss are dependent on the polarization and interparticle spacing. From Fig. S4, we can see that the gold nanodisk chain acts as a waveguide to gradually couple the LSPR with decreasing intensity along the long axis of gold nanodisk chain. The low-efficiency LSPR coupling from the initial nanodisk to neighboring nanodisks is mainly due to the propagation loss of LSPR induced by resistive heating. Such low-efficiency propagation feature of a metallic nanoparticle chain under asymmetric illumination is a big issue for the related applications in integrated optical communication.

**Supplementary References**

1. Grahn, P., Shevchenko, A., Kaivola, M. Electromagnetic multipole theory for optical nanomaterials. *New J Phys* **14**, 093033 (2012).
2. Maier, S. A., Brongersma, M. L., Kik, P. G., Atwater, H. A. Observation of near-field coupling in metal nanoparticle chains using far-field polarization spectroscopy. *Phys Rev B* **65**, 193408 (2002).
3. Maier, S. A., Kik, P. G., Atwater, H. A. Optical pulse propagation in metal nanoparticle chain waveguides. *Phys Rev B* **67**, 205402 (2003).
4. Wei, Q. H., Su, K. H., Durant, S., Zhang, X. Plasmon resonance of finite one-dimensional Au nanoparticle chains. *Nano Lett* **4**, 1067-1071 (2004).
